# Structure of a Murine Norovirus NS6 Protease-Product Complex Revealed by Adventitious Crystallisation

**DOI:** 10.1371/journal.pone.0038723

**Published:** 2012-06-07

**Authors:** Eoin N. Leen, Gabriela Baeza, Stephen Curry

**Affiliations:** Department of Life Sciences, Imperial College, London, United Kingdom; MRC National Institute for Medical Research, United Kingdom

## Abstract

Murine noroviruses have emerged as a valuable tool for investigating the molecular basis of infection and pathogenesis of the closely related human noroviruses, which are the major cause of non-bacterial gastroenteritis. The replication of noroviruses relies on the proteolytic processing of a large polyprotein precursor into six non-structural proteins (NS1–2, NS3, NS4, NS5, NS6^pro^, NS7^pol^) by the virally-encoded NS6 protease. We report here the crystal structure of MNV NS6^pro^, which has been determined to a resolution of 1.6 Å. Adventitiously, the crystal contacts are mediated in part by the binding of the C-terminus of NS6^pro^ within the peptide-binding cleft of a neighbouring molecule. This insertion occurs for both molecules in the asymmetric unit of the crystal in a manner that is consistent with physiologically-relevant binding, thereby providing two independent views of a protease-peptide complex. Since the NS6^pro^ C-terminus is formed *in vivo* by NS6^pro^ processing, these crystal contacts replicate the protease-product complex that is formed immediately following cleavage of the peptide bond at the NS6-NS7 junction. The observed mode of binding of the C-terminal product peptide yields new insights into the structural basis of NS6^pro^ specificity.

## Introduction

Noroviruses are members of the calicivirus family of positive sense RNA viruses. In humans noroviruses cause rapid onset diarrhoea and vomiting, a condition often referred to as gastric flu. Noroviral gastroenteritis is estimated to affect 21 million people annually in the United States [1], and to be responsible for up to 200,000 deaths a year in developing nations [2]. Despite the obvious medical importance of norovirus infections, there are still no vaccines or antiviral treatments.

One of the reasons for the slow progress in drug and vaccine development has been the lack of cell culture and small animal model systems for studying the molecular mechanisms and pathology of noroviral gastroenteritis. These limitations have been partly overcome thanks to the relatively recent development of cell culture [3], and reverse genetics techniques for murine norovirus (MNV) [4]–[6]. MNV is closely related to human noroviruses and some strains are known to cause gastroenteritis in STAT1 (−/−) mice [7]. The virus has therefore emerged as an important surrogate model for studying human norovirus infections at the molecular level.

The Norovirus genome is approximately 7.5 kb in length and encodes three open reading frames (ORFs). ORF2 and ORF3 encode the major and minor capsid proteins respectively [8], [9], while ORF1 encodes a non-structural polyprotein that is cleaved into functional units by the viral protease (NS6^pro^) [10]–[14]. There are five cleavage junctions in the norovirus polyprotein, so processing ultimately releases six cleavage products, all of which play essential roles in the intracellular replication cycle of the virus [12], [14]. These include NS1–2, a protein that disrupts cellular trafficking [15]; NS3, an ATPase and putative helicase [16]; NS4, a protein that also interferes with cellular protein trafficking within infected cells [17]; NS5, also known as VPg, the genome-linked protein that has key roles in the translation and replication of the viral RNA genome [18]; NS6^pro^, the viral protease itself [19]; and NS7^pol^, an RNA-dependent RNA polymerase [20].

Although there is some variation in the sequence of the five cleavage sites recognised by norovirus NS6^pro^, common features are identifiable. The amino acids on the N-terminal side of the cleaved peptide bond (the P1 position in the nomenclature of Schechter and Berger [21]) are typically glutamine or glutamic acid, while the P1′ amino acids on the C-terminal side is usually glycine or alanine; the residues at the P2 and P4 positions tend to be large and hydrophobic in nature [12], [14].

X-ray crystallographic analysis of the NS6 protease from three different strains of human norovirus [19], [22], [23] has revealed the protein to be a cysteine protease with a chymotrypsin-like fold, similar to that of the 3C proteases from picornaviruses [24], [25]. The NS6^pro^ fold is composed of two β-barrel domains tightly packed against each other; the interface between these two domains forms the peptide-binding cleft at the centre of which is the protease active site, a catalytic triad consisting of cysteine, histidine and aspartic or glutamic acid [22]. While the catalytic mechanism of NS6^pro^ and 3C^pro^ enzymes has not been fully defined, it has been shown to involve all three amino acids of the triad [22], [26]–[28] and is therefore likely to resemble the mechanism of the serine proteases.

In one of these crystallographic studies, the structure of human norovirus NS6^pro^ was determined in complex with a peptide-like inhibitor [23]. In this structure the N-terminal half of the peptide (positions P5 to P1) was covalently attached – by the action of a C-terminal Michael acceptor – to the cysteine nucleophile in the active site of the enzyme. Although the mode of binding was similar to that observed for other chymotrypsin-like proteases, the chemical modifications of the peptide that were made to add the Michael acceptor to the inhibitor resulted in steric clashes that distorted the protease active site. In this report we extend the structural work on noroviral proteases by describing the X-ray structure of full-length MNV NS6^pro^. Fortuitously the structure has been solved in a crystal form in which the C-terminus of one protease extends into the active site of another. This provides new insights into peptide recognition by MNV NS6^pro^.

## Results and Discussion

To prevent autolysis of the protease in the high concentrations used for crystallisation trials MNV NS6^pro^ was inactivated by mutation of the active site nucleophile to alanine (C139A). This mutant protease was expressed in *E. coli* and purified using affinity and size exclusion chromatography as described in the Materials and Methods. The protein crystallised by hanging drop vapour diffusion in 20% w/v PEG 3350, 0.2 M KSCN, 0.1 M Bis-Tris propane pH 7.5 to yield crystals that belong to space-group C222_1_ and have two molecules in the asymmetric unit. They diffracted X-rays to 1.6 Å resolution, producing an electron density map of extremely good quality for residues 1–123 and 132–183 in both molecules of the asymmetric unit. However, electron density for the solvent-exposed loop formed by residues 124–131 was very weak, presumably due to disorder, and so was not included in the NS6^pro^ model. The evident flexibility of this loop is consistent with previous crystallographic analyses of human norovirus NS6 proteases which found the loop to be either disordered, as in the Norwalk virus NS6^pro^ structure [22], or to adopt very dissimilar conformations, as in the structures of Chiba virus (CV) and Southampton virus (SV) NS6^pro^ [19], [23]. The final refined crystal structure of MNV NS6^pro^ has an R_free_ value of 20.8% and very good stereochemistry (full data collection and refinement statistics are summarised in Table 1).

**Table 1:** Crystallographic data collection and model refinement statistics for MNV NS6^pro^.

**Data collection**	
** Space-group**	C222_1_
** a, b, c (Å)**	50.9, 70.4, 192.3
** α, β, γ (°)**	α = β = γ = 90
** Resolution range (Å)**	48.03–1.58 (1.62–1.58)
** No. of independent reflections**	44,988
** Multiplicity** 1	12.4 (8.4)
** Completeness (%)**	94.2 (65.3)
** I/σ_I_**	29.8 (3.2)
** R_merge_ (%)** 2	4.0 (55.0)
**Model refinement**	
** No. of Non-hydrogen atoms/waters**	2611/290
** R_work_ (%)** 3	18.4 (26.6)
** R_free_ (%)** 4	20.8 (30.3)
** Average B-factor**	32.1
** RMS deviations – Bonds (Å)** 5	0.006
** RMS deviations – Angles (°)**	0.980
** Ramachandran plot**	
** Favoured**	98.3
** Allowed**	1.7
**PDB Accession Code**	4ash

1 Values for highest resolution shell given in parentheses.

2 R_merge_  = 100 ×Σ_hkl_|I_j_(hkl) − <I_j_(hkl)>|/Σ_hkl_Σ_j_I(hkl), where I_j_(hkl) and <I_j_(hkl)> are the intensity of measurement j and the mean intensity for the reflection with indices hkl, respectively.

3 R_work_  = 100 ×Σ_hkl_||F_obs_| − |F_calc_||/Σ_hkl_|F_obs_|.

4 R_free_ is the R_model_ calculated using a randomly selected 5% sample of reflection data that were omitted from the refinement.

5 RMS, root-mean-square; deviations are from the ideal geometry defined by the Engh and Huber parameters [45].

**Figure 1:**
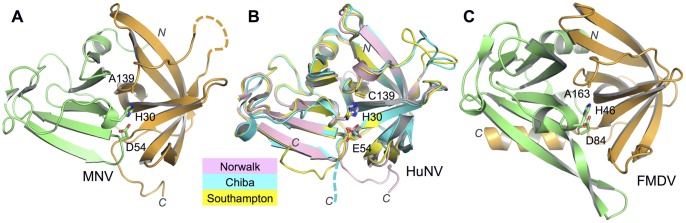
Structural comparison of the MNV NS6 protease with human norovirus NS6^pro^ and foot-and-mouth disease virus 3C^pro^. (A) Cartoon representation of the MNV NS6^pro^ structure. The N and C-terminal domains are coloured green and orange respectively. The side-chains of the amino acids that make up the catalytic triad, A139 (mutated from Cys), H30 and D54, are shown as sticks. A disordered loop formed by residues 124–130 (residues 124–131 in chain B) is indicated as a dashed line. The peptide bound to NS6^pro^ is not shown in this representation. (B) Overlay of HuNV NS6 protease structures from Chiba (PDB-ID: 1WQS), Norwalk (PDB-ID: 2FYQ) and Southampton (PDB-ID: 2IPH) viruses [19], [22], [23]. Excluding the variable C-termini, the root mean square deviations of the backbone atoms of Chiba, Norwalk and Southampton virus NS6^pro^ from MNV NS6^pro^ are 0.62, 0.43 and 0.41 respectively. The disordered C-terminus of the Chibavirus protease is shown as a dashed line. (C) Structure of FMDV 3C^pro^ (PDB-ID: 2J92) [26], coloured as in panel (A).

The overall structure of the MNV NS6 protease is nearly identical to Norwalk virus, SV and CV NS6^pro^ (Fig. 1), which comes as no surprise given the high degree of amino acid identity (∼60%) between these proteins. As described previously, the structure is an abbreviated form of the chymotrypsin-like 3C proteases from picornaviruses [19], [22], which comprise two β-barrel domains. Specifically, in norovirus NS6 proteases, the β-strands on one side of the N-terminal β-barrel are so much shorter that the domain is better considered as a single β-sheet decorated by loops (Fig. 1A). The active site triad of H30, D54, and C139 (A139 in our mutated protein) is located in the centre of the peptide-binding cleft formed at the interface of these two domains.

**Figure 2:**
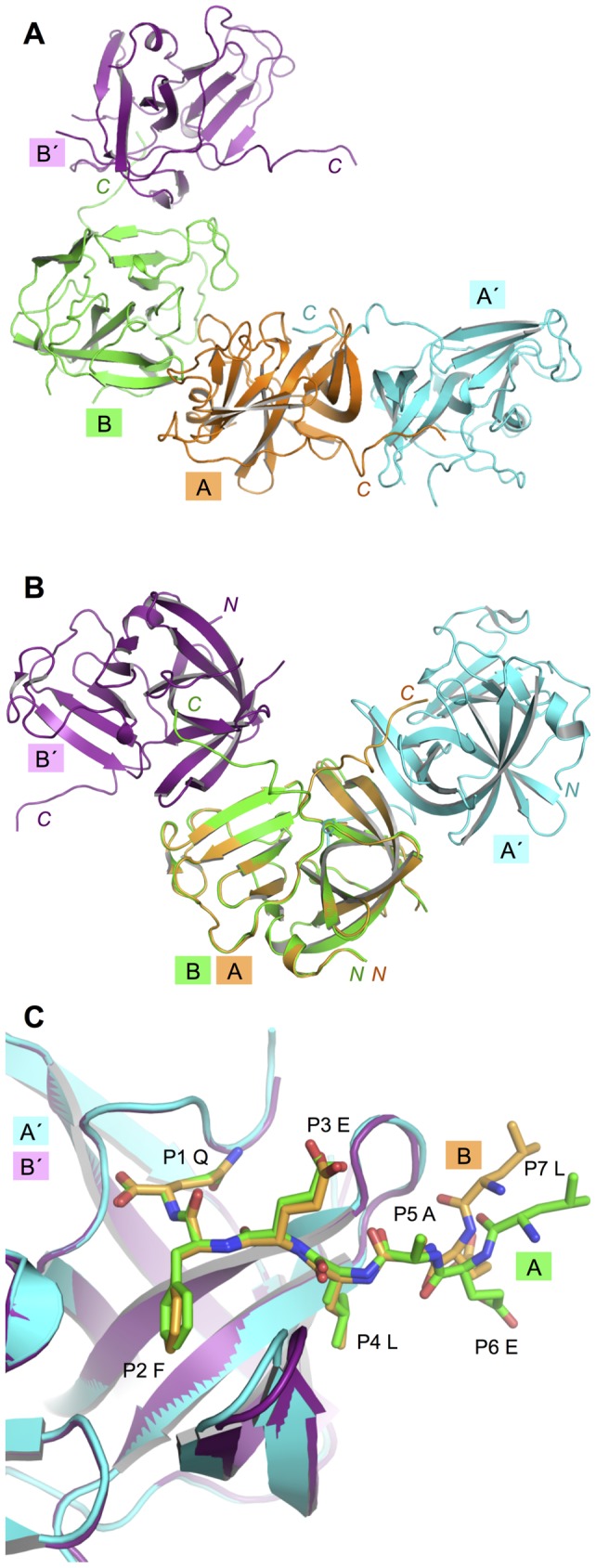
Variations in the crystal packing of the MNV NS6^pro^ A and B chains in the asymmetric unit. (A) Crystal packing of A and B chains of MNV NS6^pro^. The A and B chains of one asymmetric unit are shown along with the neighbouring molecules (labelled A' and B') into which they insert their C-termini. (B) This panel shows the same molecules that are depicted in panel A (with the same colouring) but in this case the A and B chains within the asymmetric unit are superposed; this reveals the very different contacts that they make with their closest neighbour in the crystal. (C) Here the A' and B' chains from panel A are now superposed in order to show the similarity of the conformations of the bound C-termini (shown as sticks) from the A and B chains respectively. Colour-coding is the same as panel A.

In the norovirus NS6^pro^ crystal structures published to date it has already been observed that, whereas the N-terminal helix found in picornavirus 3C proteases is retained in NS6^pro^, the C-terminal helix in 3C^pro^ is not found in the norovirus proteases [19], [22], [23]. Instead the C-terminal polypeptide of NS6^pro^ appears to be largely unstructured; it is either disordered or adopts very different conformations that appear to be determined by crystal contacts (Fig. 1B). Strikingly, in our structure the flexibility of the C-terminal polypeptide has actually allowed it to associate with the peptide-binding sites of a neighbouring molecule in the crystal – a happy accident that has revealed the structure of an MNV NS6^pro^-peptide complex (Fig. 2A). The interaction occurs for both molecules of the asymmetric unit, although in each case the C-terminus extends from the body of the protease in a different direction towards its neighbour (Fig. 2B). For chain A, the interaction is symmetric: it donates its C-terminus to a neighbouring A-chain that is related by crystallographic two-fold symmetry and binds the C-terminus received from this molecule. This pair of molecules thus forms a closed complex. In contrast, chain B donates its C-terminus to a neighbouring B-chain but binds the C-terminus of a third molecule (also a B-chain). Despite these differences, the interactions made with the neighbouring protease by the residues corresponding to the P4-P1 amino acids of the donated C-terminus are in each case essentially identical (Fig. 2C).

**Figure 3:**
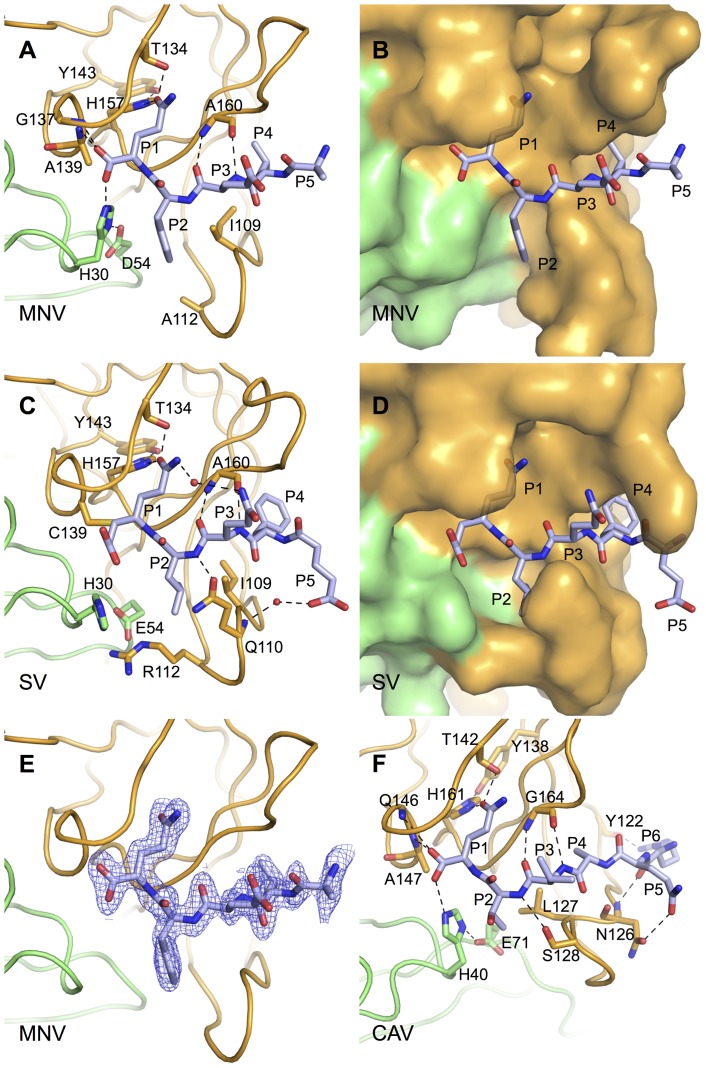
Comparative analysis of protease-peptide interactions for the P6–P1 residues in MNV and SV NS6^pro^ and CAV 3C^pro^. The N-terminal and C-terminal β-barrel domains of each protease are coloured green and orange respectively. (A) Binding of residues P5–P1 (C-terminus of NS6^pro^), shown as sticks colour-coded by atom type (Carbon – light-blue; Oxygen – red; Nitrogen – blue), within the peptide binding grove of MNV NS6^pro^. Selected side-chains from the protease are also shown as sticks. Hydrogen bonds and salt-bridges mentioned in the text are indicated by black dashed lines; all such bonds shown are ≤3.1 Å. (B) Same view as in A but showing the surface of MNV NS6^pro^. (C) Binding of residues P5–P1 from a peptide-like inhibitor to SV (a human norovirus) [23]. Water molecules involved in the protease-peptide interaction are shown as red spheres. (D) Same view as in B but showing the surface of SV NS6^pro^. (E) The refined σ-weighted 2F_o_-F_c_ map electron density (where F_o_ and F_c_ are the observed and calculated structure factors respectively) for an A-chain C-terminal peptide, shown at 1.5 σ. (F) The interaction between residues P6–P1 of a peptide ‘product’ and CAV 3C^pro^ [30].

Superposition of the crystal structure of Southampton Virus NS6^pro^ with a covalently-attached peptide-like inhibitor shows that the positions of the P4-P1 amino acids are very similar to our MNV NS6^pro^ ‘co-crystal’ structure [23] (Fig. 3A, C). The positions of the P4-P1 amino acids observed in the peptide-binding site of MNV NS6^pro^ are also very similar to the equivalent residues in the co-crystal structure of FMDV 3C^pro^:peptide complex [29] (not shown). This indicates that, although the formation of an MNV protease-peptide complex is an accident of crystallisation, crystal-packing constraints do not prevent the C-terminal residues from adopting a physiologically-relevant conformation in the binding site of a neighbouring molecule.

The adventitious insertion of the NS6^pro^ C-terminus into the peptide-binding cleft of neighbouring proteases in our crystals has provided us with a detailed view of a structure that corresponds to the protease-product complex of MNV NS6^pro^ that forms following cleavage of the NS6-NS7 junction in the viral polyprotein. All of the amino acids on the P-side of the cleavage junction are visible in the electron density map and are included in the model. The primary contacts with the protease are made by the carboxylate group of P1-Gln and side-chains of P1-Gln, P2-Phe and P4-Leu. Although the P5 and P6 amino acids make some contact with the protease, their positions differ for molecules A and B in the asymmetric unit. These differences are likely to be due to differences in crystal packing, so our structure does not give a clear indication that they are important for peptide recognition.

The co-crystal structure of SV NS6^pro^ with a P5–P1 (EFQLQ) peptide-based inhibitor covalently attached to the active site nucleophile [23] reveals a conformation for the bound peptide over the region P1–P4 that is very similar to the MNV NS6^pro^-peptide complex, although there are some differences in detail caused by sequence variation and a notable structural alteration at the active site due to the chemical modification of the peptide inhibitor needed to introduce the reactive Michaels acceptor (see below).

P1 residue recognition by MNV NS6^pro^ is mediated primarily by a specificity triad of residues composed of H157, T134 and Y143. The side-chain carbonyl of the P1-Gln in the peptide (Q183) forms hydrogen bonds with the imidazole ring of H157 and the T134 hydroxyl group (Fig. 3A); in turn, H157 is stabilised by a hydrogen bond from the hydroxyl group of Y143. This mode of interaction could be replicated by a P1-Glu residue and accounts for the ability of NS6^pro^ to cleave substrates with P1-Gln or P1-Glu. The specificity triad, which is also found in human norovirus NS6^pro^ structures, is also conserved in picornaviral 3C^pro^ enzymes from poliovirus, human rhinovirus, coxsackie A virus (CAV) and FMDV [24], [30]–[32] and explains the ability to cleave after P1-Gln *or* P1-Glu, although only FMDV is exceptional in not having a strong preference for P1-Gln [29], [33].

The P1-Gln of the peptide ‘product’ is also stabilised by interactions made by its carboxylate group with the main-chain amides of G137 and A139 (the oxyanion hole), and with the ε amine group of H30 (Fig. 3A). An essentially identical set of interactions was observed to stabilise the carboxylate group of the P1 residue in the co-crystal structure of the picornavirus CAV 3C^pro^ in complex with a peptide corresponding to the N-terminal half of the cleavage product [30] (Fig. 3F), and in the crystal structure of the Tobacco Etch virus 3C-like NIa protease where, as in our structure, the C-terminus of the protease is observed to be bound in the active site (although in the case of NIa crystals it is not known whether this is a *cis* or *trans* insertion) [34]. In contrast, this mode of interaction did not occur in the complex of SV NS6^pro^ with a peptide-like inhibitor because of the non-standard nature of the C-terminus of the SV peptide [23]; in that case the carboxylate group at the end of the peptide is separated from the C_α_ of the P1 residue by an additional C–C bond, which has the effect of distorting the geometry of the SV NS6^pro^ site (Fig. 3C). In particular, the side chain of H30 in the catalytic triad of SV NS6^pro^ is rotated out of the position needed for catalysis. In contrast, the active site geometry observed in the MNV NS6^pro^ structure is almost identical to that of the unliganded Norwalk virus protease, albeit allowing for the fact that the general acid in Norwalk virus NS6^pro^ catalytic triad is a Glu but an Asp in MNV (Fig. 1A, B) [19], [22].

**Figure 4:**
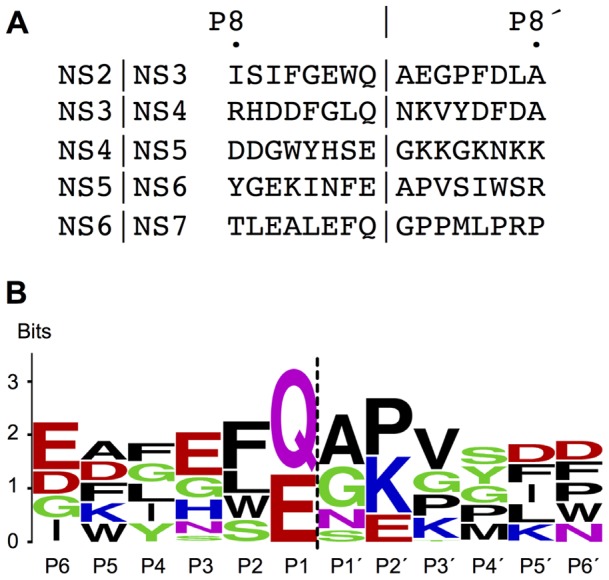
Sequence conservation of polyprotein junction in MNV that are cleaved by NS6^pro^. (A) The five cleavage junctions of MNV CW1 polyprotein (NCBI accession number YP_720001) [14]. (B) Weblogo of polyprotein cleavage junctions cleaved by MNV NS6^pro^. This Weblogo was generated using 39 MNV polyprotein sequences and the Weblogo sequence logo generator [44]. The height of the letter in each case is indicative of the degree of conservation. The Genbank accession numbers of the other sequences used to prepare the alignment are ABU55618, ABU55627, ABU55615, ABU55621, ABU55612, ABU55624, AEE10026, ABU55600, AEY83582, AEE10023, ABU55606, AEE10020, ABU55609, AEE10017, ABB02416, AEE10002, ACJ72215, AEE09999, ABU55591, AEE10005, ABU55570, AEE10008, ABU55585, AEE10014, ABU55579, AEE10011, ABU55576, ABU55597, ABU55573, ABU55603, ABU55582, ABU55594, ABU55588, ABU55567, ACS70958, ACJ72218, ABS29272, ABS29274.

The side-chain of F182 at the P2 in the MNV NS6^pro^ structure is sandwiched between H30 and I109 at the entrance to the hydrophobic S2 pocket (Fig 3A, B) but also contacts V114, V158, A159 and D54. The largely apolar nature of the S2 pocket accounts for the general selectivity for hydrophobic amino acids at the P2 amino acid in norovirus cleavage junctions [14] (Fig. 4). An exception to this is the NS4/NS5 junction of the MNV polyprotein, which has a P2-Ser. It seems unlikely that at the side-chain hydroxyl of this amino acid could interact specifically with the S2 pocket, suggesting that the NS4/NS5 junction may be a sub-optimal substrate for the protease. Intriguingly, the S2 pocket is more enclosed in human norovirus NS6^pro^ structures because the Q110-G111-R112 sequence at the top of the loop that forms one flank of the pocket is replaced in MNV NS6^pro^ by the G110-S111-A112 sequence, which has much smaller amino acids (Fig. 3A–D).

The side-chain of P3-Glu (E181) in the MNV NS6^pro^-peptide complex makes no specific interactions with the protease, though it's position is stabilised by β-strand-like interactions between its main-chain amide and carbonyl groups and the main-chain surrounding A160 (Fig. 3A), a mode of binding that is common to chymotrypsin-like proteases. The absence of any interaction with the P3 side-chain explains the diversity of residues (Q|G|K|N|E) observed at this position in MNV cleavage junctions [14], a feature that is also shared by picornavirus 3C^pro^ cleavage junctions [29], [30].

In contrast, at the P4 position in the ‘peptide’ the side-chain of L180 occupies the hydrophobic S4 specificity pocket, which is made up of T166, T161, I109, A159, I168 and I107. This mode of interaction is very similar to the binding of apolar P4 amino acids observed in structures of SV NS6^pro^ [23] and picornaviral 3C proteases [29], [35], reflecting the general – but not universal – conservation of hydrophobic functional groups at P4 in specific substrates for these enzymes [36]. In MNV cleavage junctions there is a strong preference for large hydrophobic residues at P4 (W|Y|I|F|L), all of which could be accommodated in the deep, apolar S4 pocket.

Beyond the P4 position there appears to be little specific interaction between the peptide and the protease. In MNV NS6^pro^ the side chain of the P5 residue (A179) does not contact the protease. This finding echoes the lack of observable contact between FMDV 3C^pro^ and the P5 amino acid of its peptide substrate [29]. Moreover, the high diversity of amino acid at P5 in MNV cleavage junctions (E|D|W|K|A) is consistent with the notion that this residue is not a determinant of specificity (Fig. 4). Although the P5-Glu in the co-crystal structure of SV NS6^pro^ with a peptide-like inhibitor was observed to interact with the main chain amide of Q109 via a water molecule (Fig 3C), this could be an artefact of crystallisation [23]. While it remains true that efficient catalysis by NS6^pro^ requires substrates that have at least five residues on the P-side of the scissile bond – since removal of the P5 residue from a synthetic substrate for SV NS6^pro^ resulted in a nine-fold decrease in the k_cat_/K_M_ specificity constant [23] – there is no evidence that residues beyond P4 contribute to specificity.

It is of interest to note that, despite the observation in the crystal structure of numerous specific contacts between the P4–P1 residues and the protease, comparative analysis of the proteolysis of mutated NS6^pro^ cleavage sites in the polyprotein has tended to suggest that there are relatively few strong determinants of cleavage specificity [28], [37]. Although these studies reported tolerance of substitutions at positions P4, P2, P1 and P1′, this interpretation is based on analyses of self-processing of polyprotein precursors at a *single* time point following translation in rabbit reticulocyte lysates [28] or following over-expression in *E. coli* [37]. Such techniques are lacking in sensitivity. For example, our previous work with FMDV 3C^pro^ showed that analysis of polyprotein cleavage rates by over-expression in *E. coli* could not distinguish from wild-type a mutant that in *in vitro* assays (using purified protein and synthetic peptide substrates) had only 1% of wild-type activity [26]. We suspect therefore that, consistent with the crystal structure reported here (Fig. 3) and broad patterns of sequence conservation (Fig. 4), NS6^pro^ does indeed discriminate between amino acids at the P1, P2 and P4 positions. The contribution to specificity of residues on the P′ side of the scissile bond remains to be fully investigated.

**Table 2:** Cloning and C139A mutagenic primers used in the course of the study.

MNV VPg 1–183 F	CATCATGGATCCGCCCCAGTCTCCATCTGG
MNV VPg 1–183 R	CATCATAAGCTTACTGGAACTCCAGAGCCTC
C139A QuikChange F	CTCGGGACCATCCCAGGCGAC**GCA **GGCTGTCCCTATGTTTATAAG
C139A QuikChange R	CTTATAAACATAGGGACAGCC**TGC **GTCGCCTGGGATGGTCCCGAG

Restriction sites used in cloning are underlined. Mutations introduced using QuikChange mutagenesis are in boldface.

In summary, crystallisation of MNV NS6^pro^ has provided an unexpected insight into the nature of protease-peptide interactions because of the insertion of the C-terminus of the protein (a product of NS6^pro^ cleavage) into the peptide binding cleft of neighbouring molecules in the crystal. Crystallisation has in effect captured a snapshot of the protease-product complex that forms immediately following resolution of the covalent intermediate that forms during catalysis. The protease-peptide interactions revealed in this structure provide a basis for the engineering of specific NS6^pro^ inhibitors.

It has occurred to us that by extending the C-terminus of MNV NS6^pro^ to incorporate the sequence of the NS6-NS7 cleavage junction, or to modify the sequence to that of other cleavage junctions, it may be possible to exploit the same crystal form to obtain structure of NS6^pro^-substrate complexes. This would permit examination of protease-peptide interactions on the P′ side of the cleavage junction and work towards this goal is now in progress in our laboratory.

## Materials and Methods

cDNA for MNV1 strain CW1 NS6^pro^ (a kind gift from Dr Ian Goodfellow – GenBank accession number YP724460) was amplified by PCR and ligated into the expression vector pETM11 [38], which contains a thrombin cleavable N-terminal 6x poly-histidine tag. QuikChange mutagenesis (Stratagene) was used to introduce a C139A mutation into NS6^pro^ to knock out the active site nucleophile of the enzyme. The primers used in cloning and mutagenesis are given in Table 2. The protein was expressed in BL21 (DE3) pLysS *Escherichia coli* cells by induction with 1 mM IPTG for 3 hours at 37°C. The protein was purified on TALON resin (Clontech); the polyhistidine tag was removed by overnight incubation using approximately 10 U of thrombin (Sigma-Aldrich) per milligram of purified protein. The cleaved protein was further purified by size exclusion chromatography using a Hi-Load 16/60 Superdex 75 column (GE healthcare). Peak fractions were concentrated to 750 µM in 25 mM Tris.HCl, pH 8.0 containing 200 mM NaCl and 5 mM Dithiothreitol. The purified protein contained 185 residues comprising a non-native GS sequence at the N-terminus, followed by all 183 residues of MNV NS6^pro^.

Crystals were obtained using the sitting drop vapour diffusion method by mixing 2 µL of protein solution with 1 µL of reservoir solution. This was allowed to equilibrate over 125 µL reservoir solution at room temperature. The reservoir solution was composed of 0.2 M KSCN, 0.1 M Bis-Tris propane pH 7.5, 20% w/v poly-ethylene glycol (PEG) 3350 (condition 64 in the Qiagen PACT suite of crystallisation buffers). Crystals were transferred into a cryobuffer containing 0.2 M KSCN, 0.1 M Bis-Tris propane pH 7.5, 30% w/v PEG 3350 prior to freezing in liquid nitrogen. (MNV NS6^pro^ crystals were also obtained in 30% w/v PEG 4000, 0.2 M MgCl_2_, 0.1 M Tris.HCl pH 8.5; 10% w/v PEG 8000, 8% w/v ethylene glycol, 0.1 M HEPES, pH 7.5; 20% w/v PEG 3350, 0.2 M sodium iodide, 0.1 M Bis-Tris-propane pH 6.5; and 20% w/v PEG 3350, 0.2 M sodium iodide, 0.1 M Bis-Tris-propane pH 8.5, but have not been tested for diffraction.)

X-ray diffraction data were collected at the Diamond Light Source on the I04-1 beamline (wavelength 0.917 Å) using a PILATUS 2 M detector. Data were indexed and scaled using the XDS software package [39] and phased by molecular replacement with Phaser using a weighted ensemble of the three known human norovirus protease structures as a search model [19], [22], [23], [40]. Refinement and model manipulation were performed using PHENIX and Coot respectively [41], [42]. Structure validation was performed using MolProbity [43].

## References

[1] Scallan E, Hoekstra RM, Angulo FJ, Tauxe RV, Widdowson MA (2011). Foodborne illness acquired in the United States–major pathogens.. Emerg Infect Dis.

[2] Patel MM, Widdowson MA, Glass RI, Akazawa K, Vinje J (2008). Systematic literature review of role of noroviruses in sporadic gastroenteritis.. Emerg Infect Dis.

[3] Wobus CE, Karst SM, Thackray LB, Chang KO, Sosnovtsev SV (2004). Replication of Norovirus in cell culture reveals a tropism for dendritic cells and macrophages.. PLoS Biol.

[4] Chaudhry Y, Skinner MA, Goodfellow IG (2007). Recovery of genetically defined murine norovirus in tissue culture by using a fowlpox virus expressing T7 RNA polymerase.. J Gen Virol.

[5] Ward VK, McCormick CJ, Clarke IN, Salim O, Wobus CE (2007). Recovery of infectious murine norovirus using pol II-driven expression of full-length cDNA.. Proc Natl Acad Sci U S A.

[6] Yunus MA, Chung LM, Chaudhry Y, Bailey D, Goodfellow I (2010). Development of an optimized RNA-based murine norovirus reverse genetics system.. J Virol Methods.

[7] Kahan SM, Liu G, Reinhard MK, Hsu CC, Livingston RS (2011). Comparative murine norovirus studies reveal a lack of correlation between intestinal virus titers and enteric pathology.. Virology.

[8] Jiang X, Wang M, Graham DY, Estes MK (1992). Expression, self-assembly, and antigenicity of the Norwalk virus capsid protein.. J Virol.

[9] Glass PJ, White LJ, Ball JM, Leparc-Goffart I, Hardy ME (2000). Norwalk virus open reading frame 3 encodes a minor structural protein.. J Virol.

[10] Liu BL, Viljoen GJ, Clarke IN, Lambden PR (1999). Identification of further proteolytic cleavage sites in the Southampton calicivirus polyprotein by expression of the viral protease in E. coli.. J Gen Virol 80 (Pt.

[11] Liu B, Clarke IN, Lambden PR (1996). Polyprotein processing in Southampton virus: identification of 3C-like protease cleavage sites by in vitro mutagenesis.. J Virol.

[12] Belliot G, Sosnovtsev SV, Mitra T, Hammer C, Garfield M (2003). In vitro proteolytic processing of the MD145 norovirus ORF1 nonstructural polyprotein yields stable precursors and products similar to those detected in calicivirus-infected cells.. J Virol.

[13] Blakeney SJ, Cahill A, Reilly PA (2003). Processing of Norwalk virus nonstructural proteins by a 3C-like cysteine proteinase.. Virology.

[14] Sosnovtsev SV, Belliot G, Chang KO, Prikhodko VG, Thackray LB (2006). Cleavage map and proteolytic processing of the murine norovirus nonstructural polyprotein in infected cells.. J Virol.

[15] Ettayebi K, Hardy ME (2003). Norwalk virus nonstructural protein p48 forms a complex with the SNARE regulator VAP-A and prevents cell surface expression of vesicular stomatitis virus G protein.. J Virol.

[16] Pfister T, Wimmer E (2001). Polypeptide p41 of a Norwalk-like virus is a nucleic acid-independent nucleoside triphosphatase.. J Virol.

[17] Sharp TM, Guix S, Katayama K, Crawford SE, Estes MK (2010). Inhibition of cellular protein secretion by norwalk virus nonstructural protein p22 requires a mimic of an endoplasmic reticulum export signal.. PLoS One.

[18] Schaffer FL, Ehresmann DW, Fretz MK, Soergel MI (1980). A protein, VPg, covalently linked to 36S calicivirus RNA.. J Gen Virol.

[19] Nakamura K, Someya Y, Kumasaka T, Ueno G, Yamamoto M (2005). A norovirus protease structure provides insights into active and substrate binding site integrity.. J Virol.

[20] Ng KK, Pendas-Franco N, Rojo J, Boga JA, Machin A (2004). Crystal structure of norwalk virus polymerase reveals the carboxyl terminus in the active site cleft.. J Biol Chem.

[21] Schechter I, Berger A (1967). On the size of the active site in proteases. I. Papain.. Biochem Biophys Res Commun.

[22] Zeitler CE, Estes MK, Venkataram Prasad BV (2006). X-ray crystallographic structure of the Norwalk virus protease at 1.5-A resolution.. J Virol.

[23] Hussey RJ, Coates L, Gill RS, Erskine PT, Coker SF (2010). A Structural Study of Norovirus 3C Protease Specificity: Binding of a Designed Active Site-Directed Peptide Inhibitor..

[24] Matthews DA, Smith WW, Ferre RA, Condon B, Budahazi G (1994). Structure of human rhinovirus 3C protease reveals a trypsin-like polypeptide fold, RNA-binding site, and means for cleaving precursor polyprotein.. Cell.

[25] Allaire M, Chernaia MM, Malcolm BA, James MN (1994). Picornaviral 3C cysteine proteinases have a fold similar to chymotrypsin-like serine proteinases.. Nature.

[26] Sweeney TR, Roque-Rosell N, Birtley JR, Leatherbarrow RJ, Curry S (2007). Structural and mutagenic analysis of foot-and-mouth disease virus 3C protease reveals the role of the beta-ribbon in proteolysis.. J Virol.

[27] Yin J, Bergmann EM, Cherney MM, Lall MS, Jain RP (2005). Dual modes of modification of hepatitis A virus 3C protease by a serine-derived beta-lactone: selective crystallization and formation of a functional catalytic triad in the active site.. J Mol Biol.

[28] Hardy ME, Crone TJ, Brower JE, Ettayebi K (2002). Substrate specificity of the Norwalk virus 3C-like proteinase.. Virus research.

[29] Zunszain PA, Knox SR, Sweeney TR, Yang J, Roque-Rosell N (2010). Insights into cleavage specificity from the crystal structure of foot-and-mouth disease virus 3C protease complexed with a peptide substrate.. J Mol Biol.

[30] Lu G, Qi J, Chen Z, Xu X, Gao F (2011). Enterovirus 71 and Coxsackievirus A16 3C Proteases: Binding to Rupintrivir and Their Substrates and Anti-Hand, Foot, and Mouth Disease Virus Drug Design.. J Virol.

[31] Birtley JR, Knox SR, Jaulent AM, Brick P, Leatherbarrow RJ (2005). Crystal Structure of Foot-and-Mouth Disease Virus 3C Protease: New Insights into Catalytic Mechanism and Cleavage Specificity.. J Biol Chem.

[32] Mosimann SC, Cherney MM, Sia S, Plotch S, James MN (1997). Refined X-ray crystallographic structure of the poliovirus 3C gene product.. J Mol Biol.

[33] Curry S, Roque-Rosell N, Zunszain PA, Leatherbarrow RJ (2007). Foot-and-mouth disease virus 3C protease: Recent structural and functional insights into an antiviral target.. Int J Biochem Cell Biol.

[34] Nunn CM, Jeeves M, Cliff MJ, Urquhart GT, George RR (2005). Crystal structure of tobacco etch virus protease shows the protein C terminus bound within the active site.. Journal of Molecular Biology.

[35] Matthews DA, Dragovich PS, Webber SE, Fuhrman SA, Patick AK (1999). Structure-assisted design of mechanism-based irreversible inhibitors of human rhinovirus 3C protease with potent antiviral activity against multiple rhinovirus serotypes.. Proc Natl Acad Sci U S A.

[36] Sosnovtsev SV, Hansman GS, Jiang XJ, Green KY (2010). Proteolytic cleavage and viral proteins.. Caliciviruses: Molecular and Cellular Virology.

[37] Wirblich C, Sibilia M, Boniotti MB, Rossi C, Thiel HJ (1995). 3C-like protease of rabbit hemorrhagic disease virus: identification of cleavage sites in the ORF1 polyprotein and analysis of cleavage specificity.. Journal of Virology.

[38] Zou P, Gautel M, Geerlof A, Wilmanns M, Koch MH (2003). Solution scattering suggests cross-linking function of telethonin in the complex with titin.. J Biol Chem.

[39] Kabsch W (2010). Xds.. Acta Crystallogr D Biol Crystallogr.

[40] McCoy AJ, Grosse-Kunstleve RW, Adams PD, Winn MD, Storoni LC (2007). Phaser crystallographic software.. J Appl Crystallogr.

[41] Emsley P, Cowtan K (2004). Coot: model-building tools for molecular graphics.. Acta Crystallogr D Biol Crystallogr.

[42] Adams PD, Afonine PV, Bunkoczi G, Chen VB, Davis IW (2010). PHENIX: a comprehensive Python-based system for macromolecular structure solution.. Acta Crystallogr D Biol Crystallogr.

[43] Chen VB, Arendall WB, Headd JJ, Keedy DA, Immormino RM (2010). MolProbity: all-atom structure validation for macromolecular crystallography.. Acta Crystallogr D Biol Crystallogr.

[44] Crooks GE, Hon G, Chandonia JM, Brenner SE (2004). WebLogo: a sequence logo generator.. Genome research.

[45] Engh RA, Huber R (1991). Accurate Bond and Angle Parameters for X-Ray Protein-Structure Refinement.. Acta Crystallographica Section A.

