# Correction: Structure of a Murine Norovirus NS6 Protease-Product Complex Revealed by Adventitious Crystallisation

**DOI:** 10.1371/annotation/abf34a4d-e607-4859-a7bb-91f6cfff7549

**Published:** 2012-09-11

**Authors:** Eoin N. Leen, Gabriela Baeza, Stephen Curry

There is an error in Figure 4. The correct version of Figure 4 can be seen here: 

**Figure:**
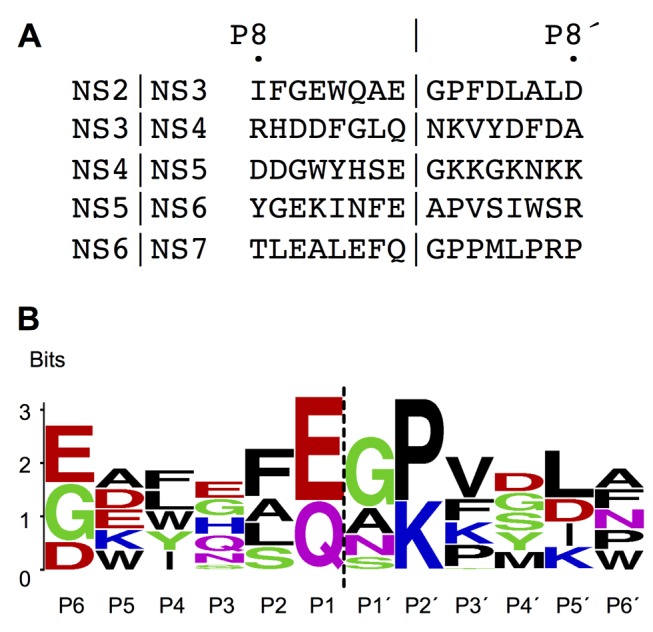


[^] 

Also, in reference to this correction, in the tenth paragraph of the Results and Discussion section, the sentence "The absence of any interaction with the P3 side-chain explains the diversity of residues (Q|G|K|N|E) observed at this position in MNV cleavage junctions [14], a feature that is also shared by picornavirus 3Cpro cleavage junctions [29], [30]" should correctly say "The absence of any interaction with the P3 side-chain explains the diversity of residues (Q|G|H|N|E) observed at this position in MNV cleavage junctions [14], a feature that is also shared by picornavirus 3Cpro cleavage junctions [29], [30]." 

